# Molecular Characterization of Putative Chordoma Cell Lines

**DOI:** 10.1155/2010/630129

**Published:** 2010-12-30

**Authors:** Silke Brüderlein, Joshua B. Sommer, Paul S. Meltzer, Sufeng Li, Takuya Osada, David Ng, Peter Möller, David A. Alcorta, Michael J. Kelley

**Affiliations:** ^1^Institute of Pathology, Ulm University, D-89081 Ulm, Germany; ^2^Executive Director, Chordoma Foundation, Durham, NC 27702, USA; ^3^Genetics Branch, National Cancer Institute, Bethesda, MD 20892, USA; ^4^Department of Medicine, Duke University, Durham, NC 27710, USA; ^5^Department of Surgery, Duke University, Durham, NC 27710, USA; ^6^Hematology/Oncology (111G), Durham VA Medical Center, 508 Fulton Street, Durham, NC 27705, USA

## Abstract

Immortal tumor cell lines are an important model system for cancer research, however, misidentification and cross-contamination of cell lines are a common problem. Seven chordoma cell lines are reported in the literature, but none has been characterized in detail. We analyzed gene expression patterns and genomic copy number variations in five putative chordoma cell lines (U-CH1, CCL3, CCL4, GB60, and CM319). We also created a new chordoma cell line, U-CH2, and provided genotypes for cell lines for identity confirmation. Our analyses revealed that CCL3, CCL4, and GB60 are not chordoma cell lines, and that CM319 is a cancer cell line possibly derived from chordoma, but lacking expression of key chordoma biomarkers. U-CH1 and U-CH2 both have gene expression profiles, copy number aberrations, and morphology consistent with chordoma tumors. These cell lines also harbor genetic changes, such as loss of p16, MTAP, or PTEN, that make them potentially useful models for studying mechanisms of chordoma pathogenesis and for evaluating targeted therapies.

## 1. Introduction

Chordomas are rare, slow-growing, and locally invasive bone tumors thought to be derived from notochordal remnants. The anatomical distribution of these tumors mirrors the location of notochord remnants, occurring most commonly at either end of the axial skeleton (32% in the clivus and 29% in the sacrum) [1]. Surgery is the mainstay of chordoma management, and radiation is commonly used as an adjuvant therapy [2]. Recurrence is common and ten-year survival is only 39% [1]. Existing chemotherapies are usually ineffective, making management of recurrences challenging [3]. Improved systemic therapies are needed; however, limited knowledge of the molecular genetics of chordoma, a small patient population, and scarcity of preclinical data have hampered efforts to develop new treatments. There are only two published clinical trials of systemic therapies in chordoma; a phase II study of 9-nitro-camptothecin [4] and a phase II study of imatinib [5]. Due to the challenge of accruing patients from this small patient population, preclinical data may need to be particularly supportive to justify undertaking future clinical trials. 

Immortalized tumor cell lines are an important cancer biology research tool, and they can be useful in predicting the clinical performance of cancer drugs [6, 7]. In particular, cell lines harboring certain drug targets present in molecularly defined cancer subtypes have proven valuable in evaluating targeted agents. Recently, molecular features that could indicate the application of targeted therapies have been identified in chordoma, including activation of the PI3K/Akt/mTOR pathway [8], activation of the IGF1R signaling cascade [9], loss of methylthioadenosine phosphorylase [9], and activation of STAT3 [10]. Chordoma cell lines with similar molecular phenotypes are potentially useful in preclinical evaluations of treatments that exploit these potential therapeutic targets. 

There are several chordoma cell lines described in the literature, but none have been fully characterized with state-of-the-art molecular techniques to evaluate their suitability as models of chordoma. Furthermore, these cell lines have never been independently validated to confirm their identity. It is well known that cross-contamination and misidentification of cell lines are a widespread problem in molecular cancer research [16, 17]. As many as one-third of cell lines originate from a different tissue or even species than claimed [18–20]. Given the scarce resources available for research on uncommon tumors, such as chordoma, the use of invalid cell lines could be catastrophic to the field. Therefore, characterization and validation of existing chordoma cell lines are of great importance to translational chordoma research. In this paper, we report our systematic characterization of cell lines reported in the literature that claim to be of chordoma origin and establish a new chordoma cell line. 

## 2. Methods

### 2.1. Cell Line Retrieval and Propagation

MEDLINE was searched for all references to chordoma cell lines using the search terms “chordoma cell line,” “chordoma cell culture,” and “chordoma AND in vitro,” and each resulting article was read to determine whether it described or reported use of a chordoma cell line. The corresponding authors of these publications were contacted, and a sample of each of their chordoma cell lines was requested, whether published or unpublished. Additionally, other unpublished chordoma cell lines were solicited from attendees of the First (2007) and Second (2008) International Chordoma Workshops and through personal communication (JS) with researchers who have published on chordoma and clinicians at medical centers with a high volume of chordoma cases. 

Once received, cells were grown to 90% confluence in 75 cm^2^ tissue culture flasks and passaged according to methods described in their respective publications or as communicated by the creators of the cell lines. The culture media, serum, and substrate used for each cell line are indicated in Table 1. All other lines were grown and passaged using methods and medias as recommended by the providers. Primary human cultures consisted of middle passage MRC-5 (American Type Tissue Culture) derived from human embryonic lung or NHF1, a gift from M. Cordeiro-S`tone, derived from newborn foreskin. 

### 2.2. Establishment of New Cell Lines 

#### 2.2.1. U-CH2

 Tumor tissue was obtained from a 72-year-old female patient with recurrent sacral chordoma. The cell line was established from tumor tissue obtained during initial surgery without neoadjuvant treatment. The patient provided informed consent. Clinical pathology determined the tumor expressed EMA (clone E29, Dako, Glostrup, Denmark), vimentin (clone VIM3B4, Dako), cytokeratin (clone AE1+AE2, Dako), and, to a minor degree, S100 protein (polyclonal, Dako). The proliferation rate determined by an anti-Ki-67 antibody (clone MIB-1, Dako) was between 5 and 10%. The tumor was minced, partially digested with collagenase, and placed in collagen-coated Primaria flasks (Falcon, Becton-Dickinson, Heidelberg, Germany) containing Iscove's modified essential medium/RPMI 1640 medium (4 : 1) (Lonza, Verviers, Belgium) with 10% fetal bovine serum (Biochrom AG, Berlin, Germany), 2 mM glutamine, and antibiotics (100 U/mL penicillin G, 100 mg/mL streptomycin). Once the cells reached confluence, they were enzymatically detached using trypsin/EDTA (0.25%/0.02%, Lonza). Cell populations with differential sensitivity to trypsin/EDTA were separated by harvesting cells after varying incubation periods. Sequential separation of trypsin-sensitive and trypsin-insensitive cells resulted in enrichment of physaliferous cells in the trypsin-insensitive population. After three passages and five months the proportion of physaliferous cells reached a steady state of approximately 30–40% of cells. Initially, U-CH2 had a doubling time of about four weeks; after 11 passages, the cells maintained a doubling time of approximately 1 week. 

#### 2.2.2. K-001

 Tumor tissue was obtained from a male patient with recurrent sacral chordoma and was cultured as described above. The patient provided informed consent under an IRB-approved study.

### 2.3. Chordoma Tissue Procurement

RNA from four chordoma tumor samples was obtained from Ardais Corporation (Lexington, MA). The patients were a 59-year-old woman with a local sacral tumor, a 52-year-old woman with a local sacral tumor, a 48-year-old man with a local tumor involving the retrorectal tissue, and a 76-year-old man with a metastasis to the arm. Tumor cells comprised 70 to 90% of the tissue sample, and no necrosis was present.

### 2.4. RNA Isolation and cDNA Generation

Media was aspirated from the flasks, cells were washed twice with PBS solution *in situ*, detached with trypsin/EDTA (Sigma catalog number T4049), and collected following two PBS washes as a cell pellet. Total RNA was purified from the pellet using the QIAGEN RNeasy mini kit (Qiagen, Valencia, CA) and QIAshredder homogenizer according to the manufacturer's instructions. Genomic DNA contamination was eliminated by DNase digestion on the column during the isolation as per Qiagen's instructions. RNA was eluted into ribonuclease-free water and immediately frozen at −80°C. Yield, concentration, and purity as 260/280 nM absorbance ratio was determined on a NanoDrop spectrophotometer (ThermoScientific, Waltham, MA). The quality of RNA for microarray was analyzed on an Agilent 2100 Bioanalyzer, and only samples with a RIN greater than 7 were used. For RNAs used for q-RT-PCR, the integrity of the RNA was confirmed by the ratio of 28S and 18S RNAs visualized under UV light after separation of denatured RNAs on 1% Tris-Borate-EDTA agarose gel. RNAs were denatured in 50% formamide, 6% formaldehyde, 10 mM sodium phosphate (pH = 7.2), and 0.5 mM EDTA by incubation at 65°C for 5 minutes, one microgram of ethidium bromide was added to the sample buffer before loading [21]. cDNA was generated from 1-2 *μ*g of total RNA using random hexamer primers and Superscript III first-strand synthesis kit (Invitrogen, Carlsbad, CA) as per the manufacturer's instructions.

### 2.5. DNA Isolation

Cultured cells were detached and collected as described above. Cell pellets were immediately frozen at −80 degrees C and processed later for genomic DNA isolation using Flexigene DNA isolation kit (Qiagen, Valencia, CA) as per the manufacturer's instructions. Concentration, size, and integrity of the DNA isolated were confirmed by NanoDrop spectroscopy, and agarose gel electrophoresis after ethidium bromide staining. 

### 2.6. Public Data Retrieval

Gene expression array data from 36 different types of normal tissue (accession GSE2361) [22] and four samples of iso-osmotically cultured intervertebral disc cells (accession GSE1648) [23] were downloaded from NCBI Gene Expression Omnibus (http://www.ncbi.nlm.nih.gov/geo/) GEO. Expression array data from 96 mesenchymal malignancies (experiment E-MEXP-353) [24] was downloaded from the European Bioinformatics Institution ArrayExpress website (http://www.ebi.ac.uk/microarray-as/ae/).

### 2.7. Gene Expression Analysis

RNA labeling and hybridization were performed by the Duke Microarray core facility as previously described [25] using the HG-U133A and HG-U133 Plus 2.0 Affymetrix GeneChip platforms, which share 22,277 identical probe sets. All arrays were background adjusted, quantile normalized, median polished, and log_2_ transformed using RMA Express [26, 27]. HG-U133A and HG-U133 Plus 2.0 arrays data were processed in two separate batches. After RMA normalization, the expression values for the 22,277 probe sets shared by the two batches were extracted and combined into a single spreadsheet. In order to compensate for variations in median signal intensity between experiments, all arrays were median centered to zero and standardized to SD = 1 [28]. Differential gene expression was assessed for each probe set using a two-tailed unequal variance *t*-test and by absolute difference in means between the chordoma set and each other set. An absolute difference in means of >2 and *P* < .001 were used as criteria for differential expression. For genes with multiple differentially expressed probe sets, the mean of the probe set values was used to represent overall gene expression. Presence or absence of selected transcripts was determined using the MAS5 algorithm (GeneChip Operating Software, Affymetrix). 

Unsupervised average linkage hierarchical clustering was performed using Cluster [29] to determine the similarity of expression among samples of a set. Two sets were examined; one set contained chordomas tumors, putative chordoma cell lines, and normal tissue (including IVD), and a second set contained chordoma tumors, putative chordoma cell lines, and mesenchymal tumors. To remove noise caused by probe sets with similar expression across all samples, clustering was only performed on probe sets with standard deviation greater than 0.7 within a sample set, which resulted in selection of 2351 probe sets for the first set of samples and 1208 probe sets for the second set. Dendrograms and heatmaps were visualized using TreeView [29].

### 2.8. Q-RT-PCR of mRNA Expression and Q-PCR of Gene Copy Number

Relative mRNA expression levels were determined using primers designed for glyceraldehydes 3′ phosphate dehydrogenase (GAPDH; NM_0002046) and the 3′ region of brachyury (T; NM_0003181) using Primer Express 3.0 (Applied Biosystems, Foster City, CA) (see Supplementary Materials for sequence of oligonucleotides available online at doi: 10.1155/2010/630129). Quantitation was performed on an ABI7900 Sequence Detection System (Applied Biosystems). Primers were validated for linear and proportional amplification using U-CH1 cDNA as a positive control for brachyury expression. For gene copy number, Brachyury-specific primers and probe were designed and used for quantitation (see Supplementary Materials). RPPH1 and TERT VIC-labeled TaqMan probes were used for normalization of input DNA (Applied Biosystems, #4403326 and #4403316, resp.) in duplex PCR reactions with Brachyury-specific primers. All samples were quantitated in quadruplicate.

### 2.9. Species Analysis

Species of origin of cell lines was determined by amplification of a segment of the aldolase gene as described previously [30]. 

### 2.10. Immunoblot

Protein extracts of cells were generated by scraping cells on ice into cold PBS containing phosphatase- and protease inhibitor cocktails (Calbiochem/EMD, Gibbstown, NJ, #524624, and #539134, resp.). Sodium dodecyl sulfate was added to a final concentration of 1%, and extracts were immediately denatured by incubation in a boiling-water bath for 5 minutes. Sample viscosity was reduced by sonication, and the protein concentration of each sample was determined using a BCA protein assay (ThermoScientific, Waltham, MA) according to the manufacturer's instructions. Five-fold concentrated Laemmli polyacrylamide gel electrophoresis (PAGE) sample buffer was added to give a final 1x concentration. Proteins were separated by PAGE, transferred to polyvinylidene difluoride membranes, and blocked in tris-buffered saline containing 10% dry milk. Brachyury, *β*-actin, cytokeratin, PTEN, phospho-AKT, and AKT were detected using commercially available antibodies Santa Cruz biotechnology (Santa Cruz, CA) #SC-17743, #SC-1616, Millipore (Billerica, MA), MAB1611, Cell Signalling (Danvers, MA) #9559, #9271, and #9272, respectively, using standard chemiluminescent detection on X-ray film.

### 2.11. Flow Cytometry

Chordoma cells were harvested from flasks with 0.05% Trypsin-EDTA, washed with PBS twice, and resuspended in 1% bovine serum albumin (Sigma, Cat# A7906, St. Louis, MO) in PBS. Cells were stained with Phycoerythrin- (PE-) labeled anti-CD24 mAb (BD Bioscience Pharmingen, Cat# 555428, San Jose, CA) and 7-AAD (Beckman-Coulter, PN IM3422, Marseille, France) for 30 min at 4°C. PE-labeled mouse IgG_1_ (BD Bioscience, Cat# 349043) was used for a negative control staining. Cells were washed with PBS twice, acquired by FACSCalibur machine (BD Bioscience) and analyzed using CellQuest software (BD Bioscience). Living (7-AAD-negative) tumor cells were analyzed for their CD24 expression.

### 2.12. Immunofluorescence

Following plating on 15 mm glass or thermanox coverslips (Thermo Scientific, Rochester NY), U-CH1 and U-CH2 cells were treated with a 3.7% formaldehyde/phosphate buffered saline (PBS) solution for 10 minutes, rinsed briefly with PBS, then permeabilized with 1% Nonidet P40 (Fluka Biochemika)/PBS for 20 minutes and followed with three five-minute PBS incubations. Brachyury was detected after sequential one-hour 37°C incubations with anti-Brachyury antibody (Santa Cruz, # SC17743) and antigoat Alexa 488 secondary antibody (Invitrogen) with three five-minute room-temperature PBS washes following each antibody incubation. Antibody dilutions were 1 : 200 and 1 : 1500, respectively. Images were obtained on an Olympus IX51 fluorescent microscope with a DP70 digital camera attachment. 

### 2.13. STR Genotyping

Genotyping was performed as previously described (Kelley, 2001) using 10 ng of cell line genomic DNA as template in each 35 cycle PCR reaction. Genetic marker analysis was performed for the eight STR markers (CSF1PO, D13S317, D16S539, D5S818, D7S820, THO1, TPOX, vWA). Seven tumor cell lines of known genotype and two samples from CEPH family 1331 were included as controls.

### 2.14. Array Comparative Genomic Hybridization

Three micrograms of DNA were fragmented by AluI/RsaI digestion and labeled by random priming incorporating cy5-dUTP (test sample) or cy3-dUTP (male reference DNA). Test sample and reference pairs were combined in Agilent hybridization buffer with Agilent blocking agent and Cot-1 DNA and hybridized to oligonucleotide CGH microarrays for 40 hours at 65°C (Agilent). For all samples, Agilent 105 k microarrays were used except for U-CH1 and U-CH2, which were hybridized to 244 k microarrays. Slides were washed according to the manufacturer's protocol, and imaged with a laser scanner (Agilent). Images were processed with Agilent Feature Extraction software and segmented in Nexus software (BioDiscovery). A threshold log_2_ ratio of  .2 or  .6 was used for gain or large gain, and −.2 and −1 were used for loss and large loss.

## 3. Results

### 3.1. Collection of Existing Cell Lines

**Figure 1:**
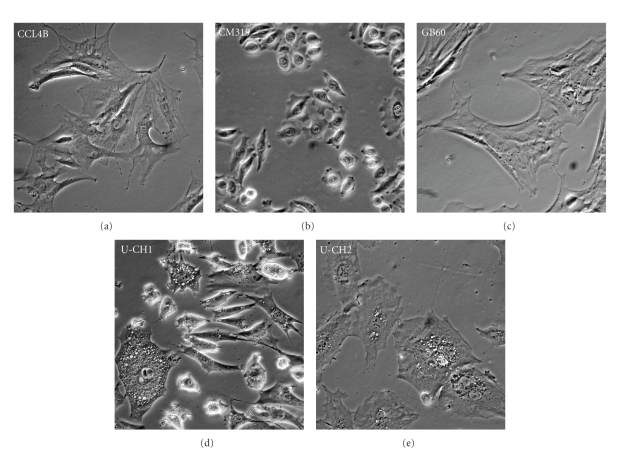
Morphological appearance of putative chordoma cell lines in culture. Phase contrast photomicrographs of cultured CCL4B, CM319, GB60, U-CH1, and U-CH2 show the chordoma tumor-like physaliphorous phenotype in only U-CH1 and U-CH2 cells.

 We identified five publications that described seven human chordoma cell lines [11–15] (Table 2). No publications describing chordoma cell lines of nonhuman origin were identified. Three cell lines were provided by Christopher Hunter (University of Calgary) [14, 15], but one line labeled CCL2 died upon arrival and was not included in any analyses. At the time this study was conducted, only one of the three cell lines described by Ricci-Vitiani et al. [13] was provided by the authors. One cell culture established at Duke University (K001) stopped growing before analysis could be completed. Images of the cell lines growing in culture are shown in Figure 1.

### 3.2. Gene and Protein Expression Analysis

**Figure 2:**
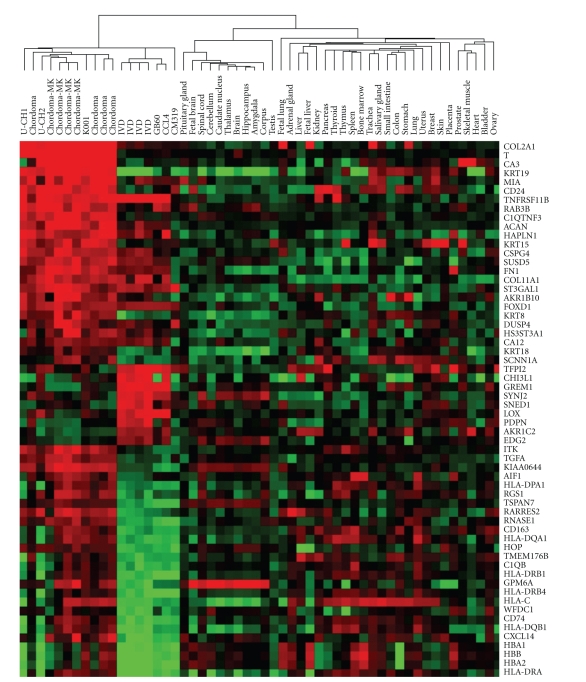
Gene expression analysis of chordoma tumors, putative chordoma cell lines, and normal tissues including intervertebral disk (IVD). Five chordoma-specific genes from among the 2351 probe sets used for cluster analysis are included; T: brachyury, KRT19: keratin19, Col2A1: collagen 2A1, CA3: carbonic anhydrase 3, and CD24. Genes with decreased relative expression are shown in green while those with increased relative expression are shown in red. Chordoma tumors (Chordoma and Chordoma-MK) and cell lines U-CH1, U-CH2, and K001 have similar expression patterns distinct from the other putative chordoma cell lines and other tissues.

**Figure 3:**
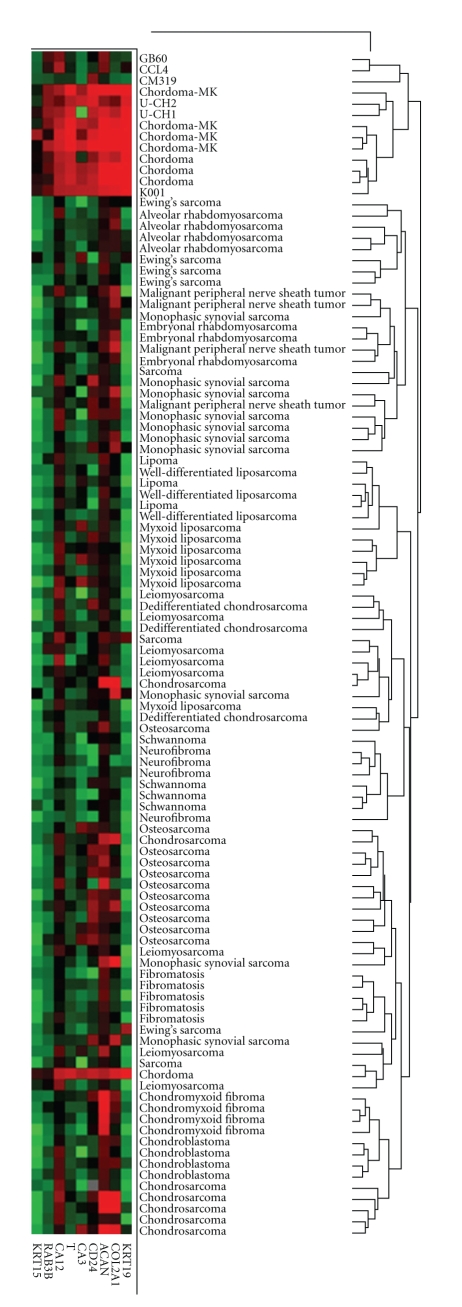
Gene expression analysis of chordoma tumors, cell lines, and mesenchymal tumors. Five chordoma-specific genes from among the 1208 probe sets used for cluster analysis are included; T: brachyury, KRT19: keratin19, Col2A1: collagen 2A1, CA3: carbonic anhydrase 3, and CD24. Genes with decreased relative expression are shown in green while those with increased relative expression are shown in red. Chordoma tumors (chordoma and chordoma-MK) and cell lines U-CH1, U-CH2, and K001 have a similar expression patterns distinct from the other putative chordoma cell lines and other tissues.

 To determine which putative chordoma cell lines originated from chordoma tissue, we compared the global gene expression profile of the cell lines with four primary chordoma tumors, a set of ninety-six mesenchymal tumors (which included four chordomas) [24], thirty-six normal tissue types [22], and four primary short-term cultured intervertebral disc samples from the central nucleus pulposus [23]. The nucleus pulpous of the IVD is the only adult tissue derived from notochordal cells, and therefore may represent a nonneoplastic analog to chordoma [21, 31]. RNA from one cell line, CCL3, did not hybridize with the array and was, therefore, not included in the gene expression analyses. Hierarchical cluster analysis of the remaining cell lines with the chordoma and normal tissues (Figure 2) showed that three cell lines, U-CH1, U-CH2, and K001, were closely clustered with the eight chordoma samples in a group distinct from cell lines GB60, CCL4, and CM-319, and the nonmalignant tissue samples. The primary bifurcation of the dendrogram separated the 36 normal non-mesenchymal tissues obtain from Ge et al. from chordoma, IVD, and cell lines, suggesting a possible batch effect. Next, we examined the expression relationship of the chordoma samples with mesenchymal tumors [32] (Figure 3). All of the chordoma tumors clustered together and away from the mesenchymal tumors except for a single chordoma tumor, which clustered near an unclassified sarcoma sample. Cell lines U-CH1, U-CH2, and K001 were again clustered closest to the remaining seven chordoma tumor samples. Three of the chordoma tumor samples that clustered with U-CH1, U-CH2, and K001 are from the same data set as the other mesenchymal tumors, thus making a batch effect unlikely as the sole explanation for the observed clustering. 

Five genes were significantly differentially expressed in chordoma tumors compared with three control groups (nonchordoma mesenchymal tumors, normal tissues, and IVD): T, CD24, COL2A1, CA3, and KRT19 (Supplementary Materials). Of the six cell lines analyzed, only K001 and U-CH2 had significant expression of all five genes. U-CH1 expressed all genes in the signature except for CA3. CCL4 expressed only KRT19 and CD24, but at levels 33- and 88-fold lower than U-CH2, respectively. CM319 expressed only CD24 and, at a very low level, COL2A1, while GB60 expressed only low levels of CD24 and COL2A1. Additional genes differentiated chordoma tumors from IVD, normal, and mesenchymal tumors individually but not in all three comparisons (Supplementary Materials). For example, KRT15 was differentially expressed in chordoma versus mesenchymal tumors and chordoma versus IVD, but not chordoma versus normal tissue. ACAN, CA12, and RAB3B were differentially expressed in chordoma versus normal tissue and mesenchymal tumors but not IVD. 

**Figure 4:**
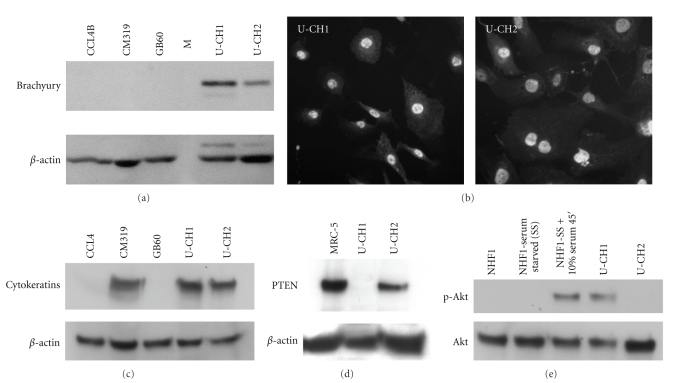
Brachyury expression in putative chordoma cell lines. (a) Immunoblot analysis of Brachyury of CCL4B, CM319, GB60, U-CH1, and U-CH2 cells reveals significant expression of the *∼*50 kDa Brachyury protein only in U-CH1 and U-CH2 cells. Molecular weight standard lane is denoted by M. (b) Immunofluorescent staining for Brachyury revealed nuclear localization of the protein in both U-CH1 and U-CH2. (c) Immunoblot analysis of cytokeratin protein expression in CCL4, CM319, GB60, U-CH1, and U-CH2. (d) Immunoblot analysis of PTEN protein expression in MRC-5 primary human fibroblasts, U-CH1 and U-CH2. (e) Immunoblot analysis of activation of Akt in normal human fibroblasts (growing, serum starved for 48 hours then with, or without, 10% added serum for 45 minutes), U-CH1 and U-CH2. Phospho-Akt- (Serine 473-) specific antibodies show Akt activation while similar protein loading was confirmed by staining for total Akt protein levels. For A, C, and E similar protein loading was confirmed by staining for *β*-actin.

To confirm the expression of Brachyury and CD24 in cell lines, we performed RT-PCR, immunoblot blot, and flow cytometry. Brachyury mRNA expression was readily detected by quantitative RT-PCR in U-CH1. Using UCH1 as a standard, Brachyury was expressed 10-fold lower in U-CH2, 160-fold lower in GB60, and below the limit of detection (less than 16,000-fold below U-CH1) in CCL4B and CM319. A human bronchial epithelial sample was included as a control and had Brachyury RNA expressed at 630-fold below U-CH1. Brachyury protein was detected by immunoblot in U-CH1 and U-CH2, but was not detected in CM319, CCL4B, or GB60 (Figure 4(a)). The level of Brachyury protein expression in U-CH1 was higher than U-CH2, consistent with the difference in mRNA expression between these lines as indicated by qRT-PCR. Immunofluorescence localized the expression of Brachyury in U-CH1 and U-CH2 to the nucleus (Figure 4(b)), similar to subcellular localization in primary chordoma tumors [24]. No staining was seen in the other cell lines. Secondary antibody alone showed no staining (data not shown). Using flow cytometry, CD24 protein was detected on the surface of three cell lines, U-CH1, U-CH2, and CM-319, but not in CCL3, CCL4b, or GB60 (data not shown). In addition, cytokeratin expression was used clinically along with other immunohistochemical markers to determine tissue of origin of tumors. Chordomas are known to express cytokeratin [33, 34]. CM319, U-CH1, and U-CH2 showed strong cytokeratin expression by immunoblot, but no expression was detected in CCL4 and GB60 cells (Figure 4(c)). 

Activation of the PI3K pathway has been implicated in chordoma pathogenesis and may be a potential therapeutic target [8, 35]. We examined expression of a key regulatory protein in this pathway, PTEN, in U-CH1 and U-CH2. As expected based on PTEN gene loss in U-CH1, no PTEN protein expression was observed (Figure 4(d)). PTEN expression in U-CH2 was near that found in normal human fibroblasts (Figure 4(d)). Heterogeneity of expression of PTEN has been previously described among chordoma tumors (Han 2009), and we observed corresponding downstream differences in phospho-Akt between these two cell lines (Figure 4(e)).

### 3.3. Array CGH Analysis

**Figure 5:**
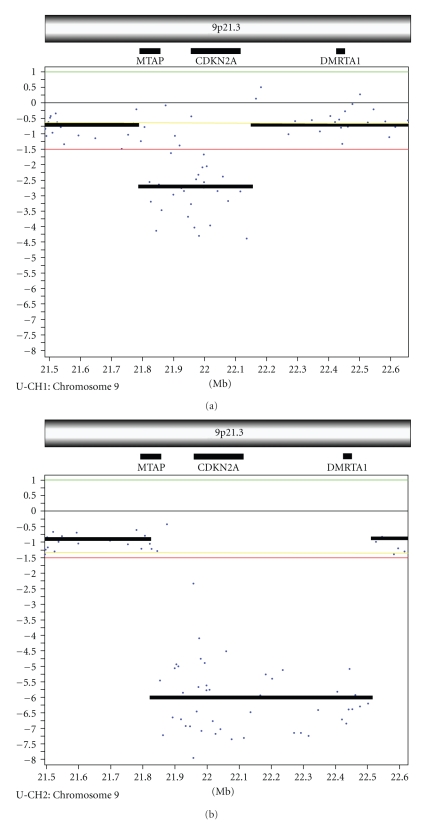
Chromosome 9p21.3 region from array CGH analysis of U-CH1 (a) and U-CH2 (b) cell lines. Both cell lines show loss of the 9p21.3 region encompassing CDKNA2, encoding the cyclin dependent kinase inhibitor p16, MTAP, encoding methylthioadenosine phosphatase, and for U-CH2, DMRTA1, encoding the transcription factor DMRT-family A1 protein. Each dot represents the copy number for the array probe for that region, and a line shows the copy number averaged across the region. Relative signal intensity values are shown on the y-axis; threshold log_2_ ratio of  .2 or  .6 was used for gain or large gain, and −.2 and −1 were used for loss and large loss.

 aCGH analysis was performed on six cell lines to determine whether their copy number aberrations (CNAs) were consistent with that of chordoma tumors [11, 36]. Cell line CCL3 did not hybridize to the human aCGH array in two separate attempts and was not further characterized for CNAs. No CNAs were detected in cell lines CCL4 and GB60 while U-CH1, UCH-2, and CM-319 had multiple CNAs (Supplementary Materials), suggesting that only U-CH1, U-CH2, and CM-319 are derived from tumor cells. Both U-CH1 and U-CH2 showed biallelic loss of the CDKN2A and CDKN2B loci on chromosome 9p21 (Figure 5). This deletion also encompassed the entire MTAP locus in U-CH1 and is deleted to near the MTAP locus in U-CH2. CNAs within this chromosomal region in U-CH1 and U-CH2 were confirmed by PCR amplification using primers at the 5′ and 3′ ends of the MTAP gene, and within the CDKN2A and DMRTA1 genes (Supplementary Materials). Both the Rb locus on chromosome 13 and the PTEN locus on chromosome 10 had loss in U-CH1, but not U-CH2 (data not shown). Consistent with this result, the PTEN transcript was not detected by gene expression microarray in U-CH1, but was present in U-CH2 and the other cell lines and tumors (Supplementary Materials). No chromosomal regions of high-level amplification were found. U-CH1 had numerous CNAs with losses on chromosomes 1, 3, 4, 9, 10, 11, 13, 18, and 22, as well as gains on chromosomes 1,7,9,14,15,17,18, and 19 (Supplementary Materials). U-CH2 had losses on 1p, 2, 3, 4, 8,10,14,16,17,19, 20, and X; gains were observed on 1, 6, 7, 12, 15, 16, 19, and X (Supplementary Materials).

### 3.4. Brachyury Copy Number

 We recently described an increase in copy number of the Brachyury gene, *T*, in families with predisposition to chordoma [37]. We, therefore, performed quantitative PCR using a *T* gene exon 6 probe on cell lines U-CH1 and U-CH2 that were found by aCGH to have multiple CNAs. aCGH showed CNA in the region of *RPPH1*, which is often used as a control in qPCR; therefore, we used *TERT* as the control, which did not appear to have CNA on aCGH. U-CH1 showed qPCR relative value of 1.11 (range: 0.93 to 1.40) compared to *TERT*, indicating that U-CH1 does not have an extra copy of the *T* gene, and consistent with the aCGH that shows no gain in the 6q region. U-CH2 had values of 1.36 (range: 1.28 to 1.53), suggesting U-CH2 has three copies of T, which is consistent with aCGH showing gain on the telomeric end of 6q (Supplementary Materials).

### 3.5. Species Analysis

**Figure 6:**
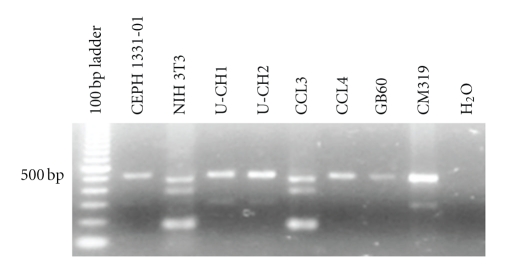
Species analysis of putative chordoma cell lines. Agarose gel electrophoresis of ethidium-stained DNA of aldolase PCR analysis of genomic DNA reveals murine origin for CCL3 as shown by similar amplicon fragment sizes found in the murine cell line, NIH 3T3, as compared to those generated from the normal human genomic sample, CEPH 1331-01. The other cell lines (CCL4, CM319, GB60, U-CH1, and U-CH2) gave similar amplicon sizes as those for the human control genomic sample CEPH 1331-01.

Because CCL3 did not hybridize with either the expression or the aCGH array, we considered that it might not be of human origin. To determine the species of origin, we used PCR to amplify a segment of the aldolase gene that results in species-specific PCR product lengths [30]. An Epstein-Barr virus transformed lymphoblast genomic DNA sample from a CEPH family individual, CEPH 1331-01, was used as a control, and it generated the expected 500 and 300 bp fragments while CCL3 and a mouse genomic DNA sample from cultured NIH-3T3 mouse cells resulted in an identical pattern of a predominant 180 bp fragment and two larger faint bands (Figure 6). All other cell line samples gave similar amplicons as found for CEPH 1331-01, thus confirming their human origin. Sequence analysis of the 180 bp fragment from CCL3 confirmed its origin from mice.

### 3.6. Genotyping

 Because cell line identity has often been confused in laboratories, we performed genotyping of eight STR markers from the CODIS panel for cell lines U-CH1, U-CH2, and CM-319 (Table 3), as well as CCL4 and GB60 (data not shown). The genetic profile of these lines was not found in the ATCC STR genotype database of cell lines, indicating they are not derived from common existing cell lines.

## 4. Discussion

Based on our comprehensive characterization, only one of five chordoma cell lines previously described in the literature has molecular, genetic, and morphological features typical of chordoma. We also report the creation of a new cell line, called U-CH2, which exhibits chordoma-like characteristics. An additional cell line, K001, was grown for over a year in culture and maintained chordoma-like gene expression patterns, but has since stopped growing and is no longer available. 

One putative cell line, CCL3, was found to be of murine origin. All other cell lines were confirmed to be human and have genotypes distinct from those of commonly used cell lines in the ATCC repository indicating that they are likely to be independently derived. However, neither GB60 nor CCL4 have genetic alterations typical of human cancer, suggesting they may be immortalized clones of nonmalignant cells. At a minimum, because all chordomas previously analyzed by array CGH harbored multiple copy number abnormalities [36], the lack of copy number aberrations indicates that GB60 and CCL4 are unlikely to be chordoma tumor cells. It is possible that these cell lines originate from stromal cells involved in the tumors from which they were cultured. Furthermore, only U-CH1 and U-CH2 maintain the physaliferous cell morphology typical of chordoma tumors. 

Hierarchical clustering revealed that the global gene expression profiles of U-CH1, U-CH2, and K001 are highly similar to that of chordoma tumors, while the remaining cell lines clustered apart from chordoma tumors. In addition, we identified five genes that were significantly differentially expressed between chordoma tumors and 92 other mesenchymal tumors, 36 normal tissue types, and intervertebral disc (IVD) tissue. All five of these genes were highly expressed in K001 and U-CH2, four were highly expressed in U-CH1, one was weakly expressed in CM319, and none were expressed in GB60 or CCL4. The protein products of two of these genes, *T* (Brachyury) and CD24 (HSA, heat stable antigen), are used as diagnostic markers of chordoma [38] and were therefore measured using biochemical approaches. Brachyury protein was detected only in U-CH1 and U-CH2, while CD24 protein was detected in U-CH1, U-CH2, and CM319.

Based on these observations, we conclude that only U-CH1 and U-CH2 faithfully maintain the morphology, gene expression profile, and genomic alterations characteristic of chordoma tumors. CM319 does not appear physaliferous and does not express most genes typically expressed by chordoma tumors; however, it has abnormal genetics consistent with being a cancer cell line and expresses CD24. We, therefore, cannot conclusively rule out the possibility that it was derived from chordoma cells. It is conceivable that CM319 comes from a dedifferentiated or atypical chordoma, or that its gene expression changed significantly in culture. On the other hand, CD24 is expressed at the surface of cellular subsets of neural lineage differentiation [33] and has been reported in a number of tumor types [39], so this cell line could have originated from a tumor other than chordoma, or could have become contaminated with another cancer cell line that has not been genotyped by ATCC. To avoid misidentification of chordoma-like cell lines (U-CH1 and U-CH2) in future publications, we recommend genetic verification of cell line identity using the genotype information reported here.

We found that three cell lines reported in the literature, CCL3, CCL4, and GB60, are either contaminated or established from nonchordoma cells. CCL4 and GB60 may indeed be derived from cell populations contained in a chordoma tumor, but are most likely not neoplastic chordoma cells. Use of these cell lines has already been reported in several publications [10, 13–15, 40]. Our findings call into question the relevance of results generated using these cell lines to chordoma. 

Because chordoma cells are very slow-growing (doubling time >5 days), chordoma cell cultures may be particularly susceptible to overgrowth by immortalized stromal cells or contamination with fast-growing cell lines. Thus, we propose the following criteria for determining whether a cell line is of chordoma origin. 

### 4.1. Morphology

 Chordoma tumors are comprised of morphologically heterogeneous cells, including vacuolated physaliferous cells, small stellate cells, and various other cell types [35]. Others have reported that chordoma cell cultures reflect a similar degree of morphological heterogeneity, with the proportion of physaliferous cells changing over time [41, 42]. In addition to the cell lines reported herein, we have attempted to culture five other chordoma tumors, and, in each case, over time the physaliferous cells were replaced by fusiform or spindle-like cells before the culture ultimately stopped growing. It is not clear whether the physaliferous cells changed in appearance, or whether the fusiform cell populations outgrew the physaliferous cells. Furthermore, it is unknown which cell population(s) represent the neoplastic cells driving the tumor. Murad and colleagues found mitoses exclusively in the stellate cell population, and suggested that stellate cells are the primary neoplastic cells, perhaps differentiating into physaliferous cells [43]. Indeed, dedifferentiated chordomas, which have much higher mitotic activity than conventional chordomas, are characterized by an absence of physaliferous cells [8]. U-CH1 and U-CH2 consistently maintain approximately 30%–50% physaliferous cells. Based on our observations and the morphological heterogeneity of chordoma cells *in vivo*, we do not believe that the absence of physaliferous cells alone should exclude a cell line from being considered of chordoma origin; conversely, the presence of physaliferous cells, a hallmark of chordoma, should be a strong positive indicator of a chordoma cell line.

### 4.2. Genetics

 Comparative genomic hybridization (CGH) and array CGH analyses have confirmed that chordomas are characterized by complex genomes, uniformly possessing multiple copy number changes and rearrangements [11, 36]. Therefore, chordoma cell lines should also be expected to uniformly harbor multiple genetic alterations, and cell lines with no or few genetic alterations are unlikely of chordoma origin. A cell line is expected to have similar genetic changes as the originating chordoma tumor. As with other cell lines, the unique identity of putative chordoma cell lines can be determined by genotyping, and we recommend the panel of STR markers used by ATCC. Ideally, the genotype of a cell line should be demonstrated to be the same as that of the patient from whom the tissue used to derive the line was obtained.

### 4.3. Gene and Protein Expression

 Of the five genes we identified to be significantly overexpressed in chordoma, Brachyury, a transcription factor present in the nucleus of notochord cells [44], is the most specific for chordoma [24]. By immunohistochemistry, Brachyury is highly expressed in chordoma but not in a wide variety of normal or neoplastic tissue, and is, therefore, used as a diagnostic marker for chordoma [24]. Other than chordomas, only hemangioblastomas highly express Brachyury [45, 46]. However, recently Palena and colleagues reported modest Brachyury expression in a number of carcinomas and carcinoma-derived cell lines [47]. Therefore, the presence of Brachyury alone is not sufficient to indicate the validity of a chordoma cell line. Conversely, one study found that only 89% of chordomas expressed Brachyury [38], suggesting that the absence of Brachyury expression does not rule out the validity of a chordoma cell line. Furthermore, the chondrocytic differentiation potential of chordoma cells has been previously reported, and it has been suggested that some chordomas undergo differentiation that mimics the development of the nucleus pulposus [48]. Our analysis, and work of Vujovic and colleagues [24], demonstrated that Brachyury is not expressed in nucleus pulposus cells which are derived from the notochord [31], suggesting that cells of notochordal origin, and presumably chordoma, can lose Brachyury expression.

For cell lines that lack Brachyury but have abnormal genetics (like CM319), expression of one or more markers of chordoma including CD24, collagen type II alpha 1, keratin 19, and carbonic anhydrase 3 could offer evidence of chordoma-derived cells. If material from the original tumor is available, expression of these genes should be compared between the tumor and cell line to determine if expression levels changed in vitro. Cluster analysis of global gene expression may be required to further define the likely origin of a non-Brachyury expressing cell line. 

Our study has several limitations. First, we were unable to compare the cell lines with the tumors from which they were derived. Furthermore, we are unable to apply a tissue of origin analysis because the normal tissue from which chordoma is thought to be derived, the notochord, is not available to us for expression analysis. However, our cluster analysis demonstrates that the cell lines U-CH1, U-CH2, and K001 are likely to share the same tissue of origin. Second, all existing cell lines have been derived from chordomas arising in the sacrum. Indeed, the majority of biological analyses of chordoma have been done on sacral chordoma, which present as much larger tumors. Thus, it is unknown whether chordomas arising in clivus or other areas can be modeled by chordoma cell lines of sacral origin.

## 5. Conclusions

In summary, two cell lines, U-CH1 and U-CH2, have characteristics consistent with chordoma origin and are available for use as models of chordoma. One or both of these cell lines have molecular properties that could model specific therapeutic targets such as p16 deletion [36], MTAP deletion [9], PTEN inactivation [8, 49], and Brachyury gene duplication [37]. Published results using the other cell lines studied may not be relevant to human chordoma.

## Supplementary Material

The sequence of oligonucleotides used for quantitative PCR and quantitative RT-PCR are indicated in Section 1.
Section 2 contains a listing of genes whose expression in microarray datasets differentiates chordoma tumor samples from either non-chordoma mesenchymal tumors, normal tissues, or intervertebral disc. Some genes differentiate chordomas from more than one non-chordoma group.
Section 3 displays aCGH data graphically for each of the putative chordoma cell lines studies (CCL4, CM319, GB60, U-CH1, U-CH2). Green shading within or on right side of the chordomosomal ideogram represents extra copies while red shading within or on left side represents copy number loss. CCL4 and GB60 have essentially no copy number aberrations while CM319, U-CH1, and U-CH2 have multiple copy number aberrations (though no high level gains), consistent with origin from chordoma.
Section 4 shows that PCR of genomic DNA from U-CH1 and U-CH2. Lack of amplification of a PCR product confirms deletion of p16 in both cell lines.
Section 5 tabulates array expression levels of PTEN, Vimentin and Keratin 8/19 in cell lines and select chordoma tumor samples. Microarray data was analyzed with MAS5 and scored as present (P) or absent (A) and a level of expression calculated. All four probe sets for PTEN showed absent expression in U-CH1, while at least two indicted expression of PTEN in all other cell lines and tumor samples. The single probe set for vimentin included on all chips indicated expression in all cell lines and tumor samples. Keratins 8 and 19 were variably present depending on the probe set but at least one probe indicated expression in all samples.

## Figures and Tables

**Table 1:** Publications of human chordoma cell lines and their growth media.

Cell line	Reference	Obtained from author	Growth medium
U-CH1	[11]	yes	IMDM/RPMI (4 : 1) 10% FBS collagen-coated flasks
CM-319	[12]	yes	RPMI 1640 10% FBS
GB60	[13]	yes	DMEM/F12 10% FBS
(Unnamed in manuscript)	[13]	no	n/a
(Unnamed in manuscript)	[13]	no	n/a
CCL3	[14]	yes	DMEM/F12 10% FBS
CCL4	[15]	yes	DMEM/F12 10% FBS
U-CH2	Unpublished	yes	IMDM/RPMI (4 : 1) 10% FBS collagen-coated flasks
K001	Unpublished	yes	IMDM/RPMI (4 : 1) 10% FBS collagen-coated flasks

**Table 2:** Characteristics of cell lines.

Cell line	Growth pattern	Expression array pattern	aCGH	Brachyury expression	CD24 expression
U-CH1	Physaliferous	Chordoma-like	Multiple CNV	Positive	Positive
U-CH2	Physaliferous	Chordoma-like	Multiple CNV	Positive	Positive
K001*	Physaliferous	Chordoma-like		Positive	Positive
CCL3**	Spindle	Inadequate	Inadequate		Negative
CCL4	Spindle	Not chordoma-like	Normal	Negative	Negative
GB60	Spindle	Not chordoma-like	Normal	Low RNA; negative protein	Negative
CM-319	Polygonal	Not chordoma-like	Multiple CNV	Negative	Positive

*K001 was partially characterized before the cell line was lost

**CCL3 is of nonhuman origin

IVD = intervertebral disc tissue

**Table 3:** STR Genotype of Three Putative Chordoma Cell Lines.

Cell line	CSF1PO	D13S317	D16S539	D5S818	D7S820	THO1	TPOX	vWA
U-CH1	10,11	11,13	12,13	11,12	9,12	7	8,11	17
U-CH2	11,12	11	12	10,11	8,12	6,9.3	8	17
CM-319	9,10	12,13.3	9,10	11,12	8,12	7	8,12	16,18
