# Supplementary material for: Molecular Characterization of Putative Chordoma Cell Lines
Source: Sarcoma. 2010 Dec 30;2010:630129. doi: 10.1155/2010/630129 (PMC3022207; doi:10.1155/2010/630129)
Supplement: Supplementary file 1 — The sequence of oligonucleotides used for quantitative PCR and quantitative RT-PCR are indicated in Section 1. Section 2 contains a listing of genes whose expression in microarray datasets differentiates chordoma tumor samples from either non-chordoma mesenchymal tumors, normal tissues, or intervertebral disc. Some genes differentiate chordomas from more than one non-chordoma group. Section 3 displays aCGH data graphically for each of the putative chordoma cell lines studies (CCL4, CM319, GB60, U-CH1, U-CH2). Green shading within or on right side of the chordomosomal ideogram represents extra copies while red shading within or on left side represents copy number loss. CCL4 and GB60 have essentially no copy number aberrations while CM319, U-CH1, and U-CH2 have multiple copy number aberrations (though no high level gains), consistent with origin from chordoma. Section 4 shows that PCR of genomic DNA from U-CH1 and U-CH2. Lack of amplification of a PCR product confirms deletion of p16 in both cell lines. Section 5 tabulates array expression levels of PTEN, Vimentin and Keratin 8/19 in cell lines and select chordoma tumor samples. Microarray data was analyzed with MAS5 and scored as present (P) or absent (A) and a level of expression calculated. All four probe sets for PTEN showed absent expression in U-CH1, while at least two indicted expression of PTEN in all other cell lines and tumor samples. The single probe set for vimentin included on all chips indicated expression in all cell lines and tumor samples. Keratins 8 and 19 were variably present depending on the probe set but at least one probe indicated expression in all samples. [file 630129.f1.pdf]

Supplementary Material

1. Sequence of oligonucleotides used for quantitative PCR
2. List of genes distinguishing chordoma samples from other samples
3. Images of aCGH (global)
4. Genomic PCR for 5’ MTAP, 3’ MTAP, p16 and DMRTA1 CNA
5. Microarray expression levels of PTEN, vimentin and keratin 8/19 in cell lines and select chordoma tumor samples.

.

1. Sequences of Oligonucleotides used for quantitative PCR

For qRT-PCR of mRNA expression:

Brachyury (T):	Forward	TGG CAG TCT CAG GTT AAG AAG GA
	Reverse	AGG TGT GAG CAA GGG ATG CT
GAPDH:	Forward	GACCCCTTCATTGACCTCAACTAC
	Reverse	GCTCCTGGAAGATGGTGATGG

For q-PCR of genomic DNA

Brachyury (T):	Forward	GTA CTC CCA ATG TAC GGT TTG TTG
	Reverse	TCA GCA AGT CTA GTC CCG ATG AC
	Probe	6-Fam-CTCTGTCATGTCATTCTG-MGB

2. List of genes distinguishing chordoma samples from other samples.

Gene	Mesenchymal Tumors	Normal Tissues	Intervertebral Disc
COL2A1	x	x	x
T	x	x	x
KRT19	x	x	x
CA3	x	x	x
CD24	x	x	x
ACAN	x	x	
CA12	x	x	
RAB3B	x	x	
KRT15	x		x
KRT18	x		
AKR1B10	x		
KRT8	x		
SCNN1A	x		
MIA		x	
HAPLN1		x	
COL11A1		x	
SUSD5		x	
FN1		x	
C1QTNF3		x	
CSPG4		x	
TNFRSF11B		x	
DUSP4		x	
ST3GAL1		x	
HS3ST3A1		x	
FOXD1		x	
HLA-DQB1			x
KIAA0644			x
ITK			x
TGFA			x
KIAA0644			x
RARRES2			x
RNASE1			x
RGS1			x
AIF1			x
CXCL14			x
HOP			x
HLA-C			x
HLA-DRA			x
HLA-DPA1			x
GPM6A			x
HLA-DQA1			x

CD74	x
TSPAN7	x
CD163	x
HLA-DRB1	x
WFDC1	x
SYNJ2	x
HLA-DRB4	x
TMEM176B	x
C1QB	x
LOX	x
CHI3L1	x
SNED1	x
PDPN	x
TFPI2	x
HBA1	x
HBA2	x
HBB	x
EDG2	x
AKR1C2	x
GREM1	x

3. Images of aCGH (global) for putative chordoma cell lines. Green within or on right side of ideogram represents extra copies while red within or on left side represents copy number loss.

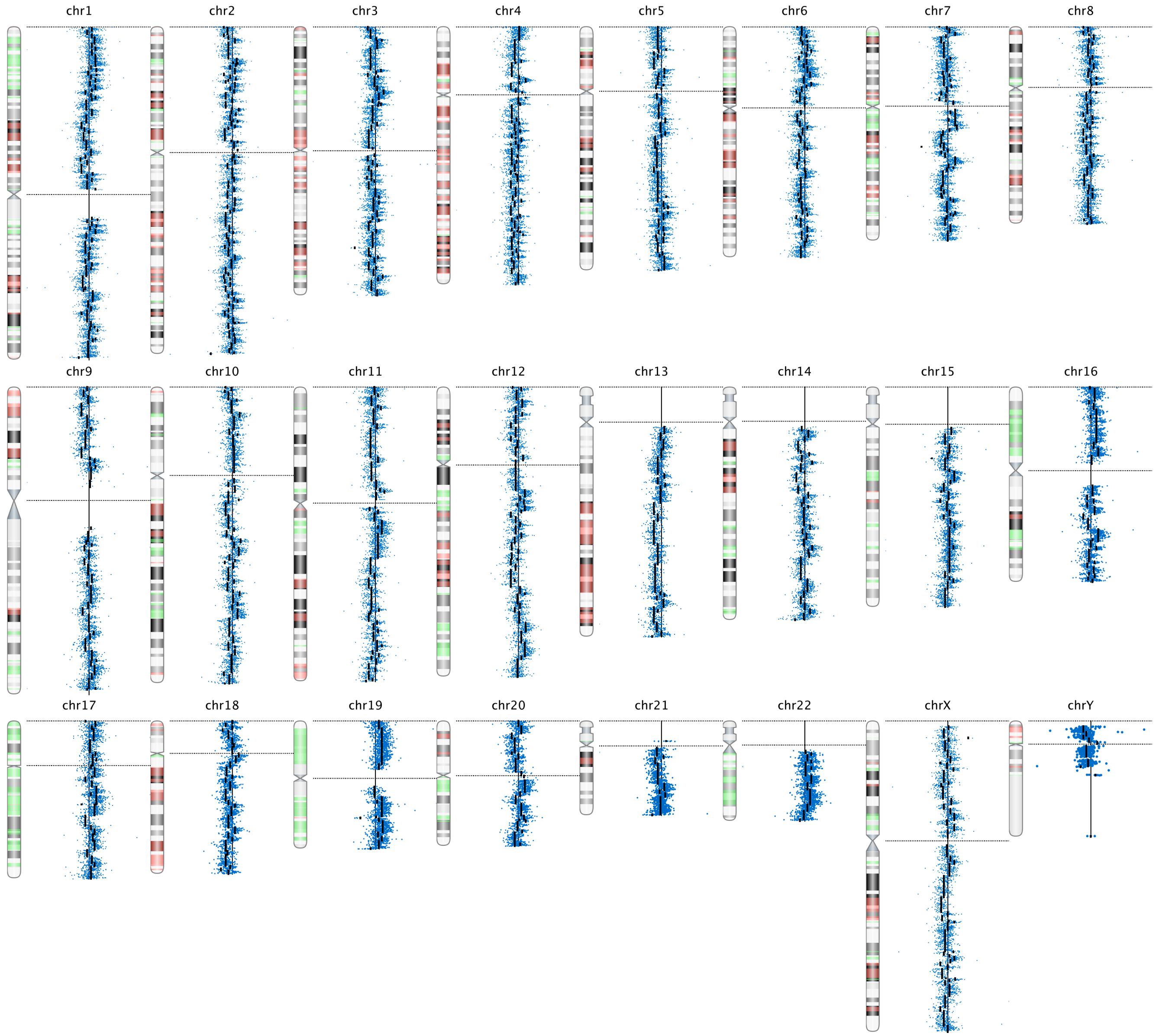

Sample: CCL4

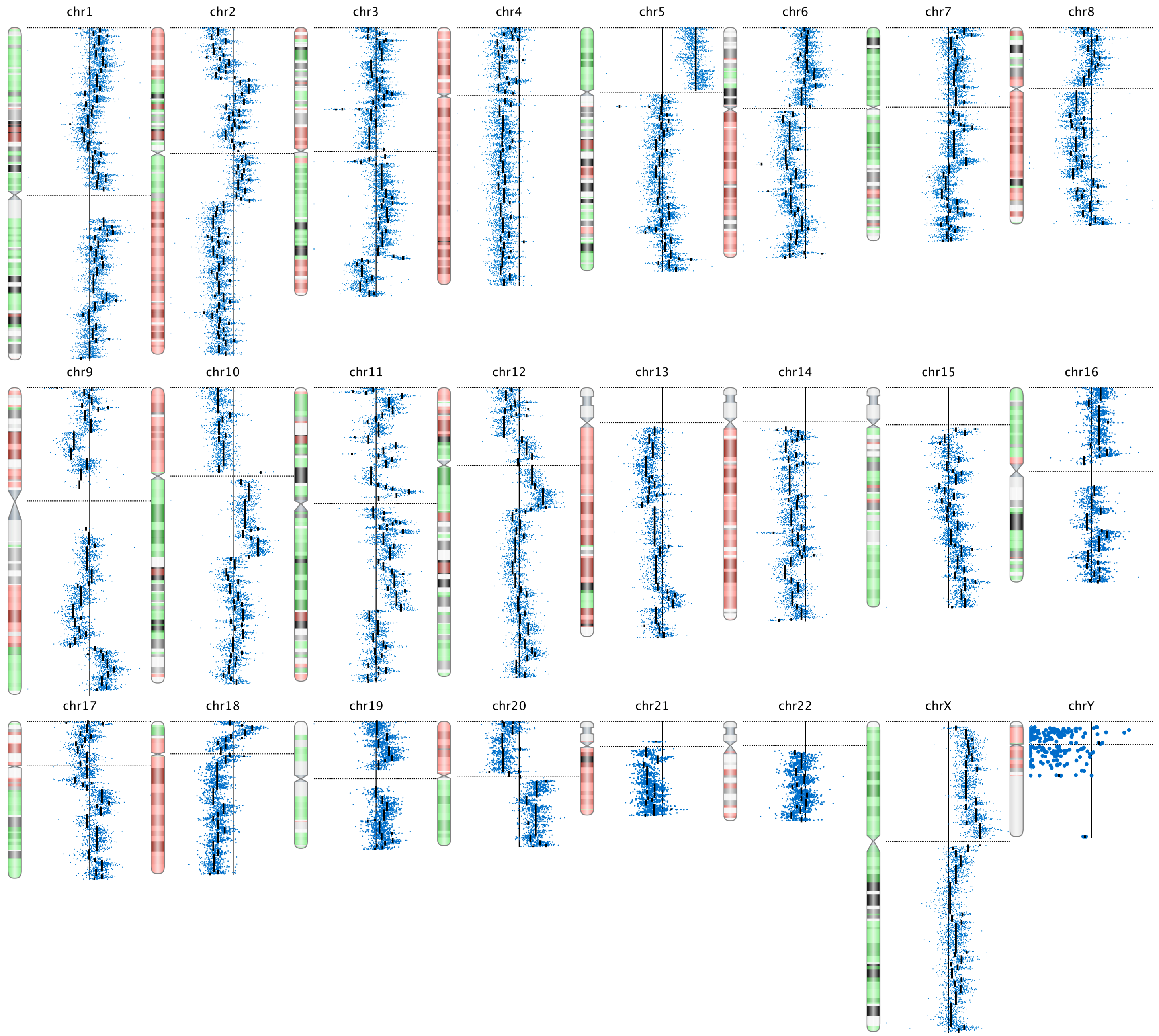

Sample: CM319

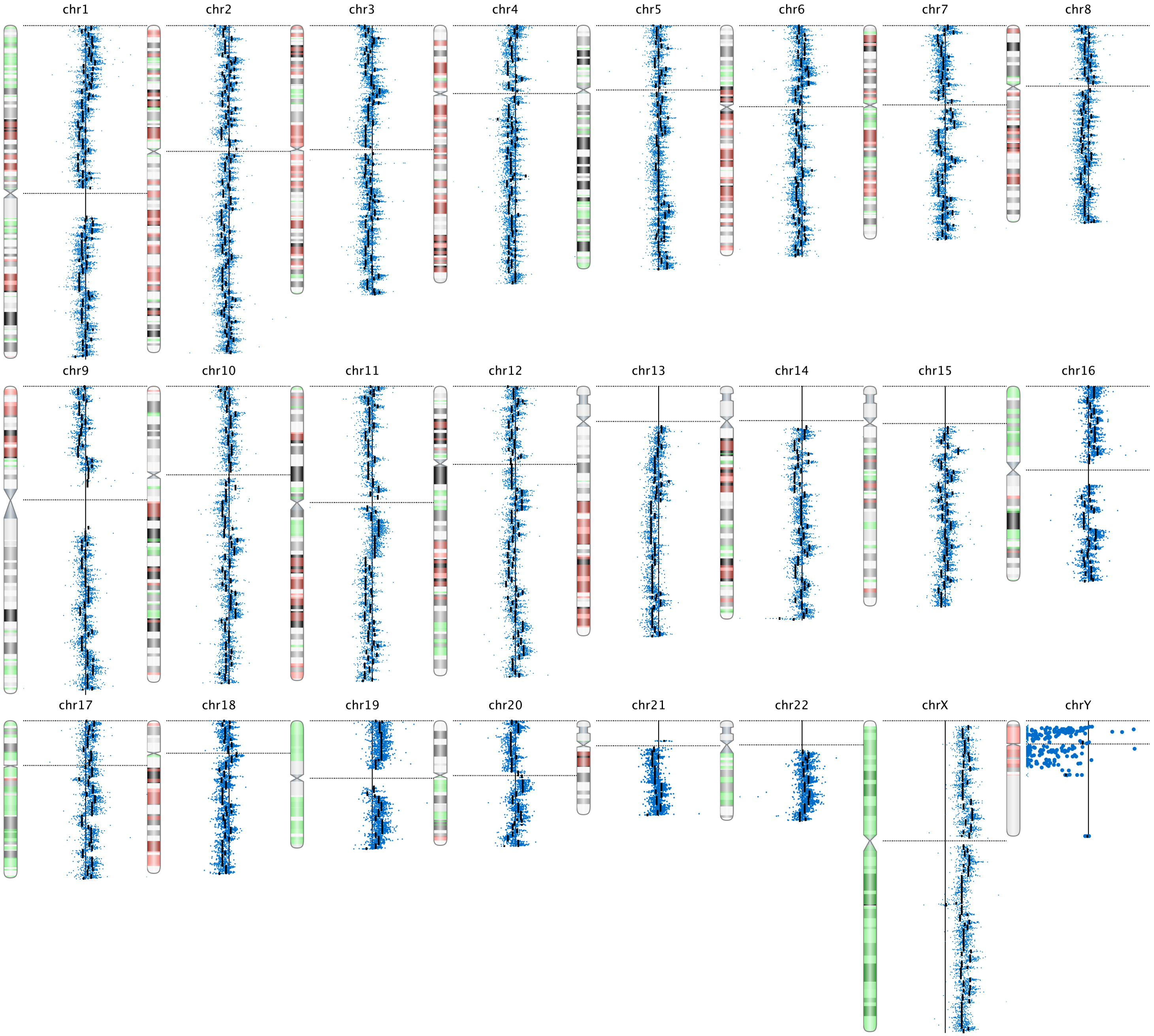
chr1
chr2
chr3
chr4
chr5
chr6
chr7
chr8
chr9
chr10
chr11
chr12
chr13
chr14
chr15
chr16
chr17
chr18
chr19
chr20
chr21
chr22
chrX
chrY

Sample: GB60

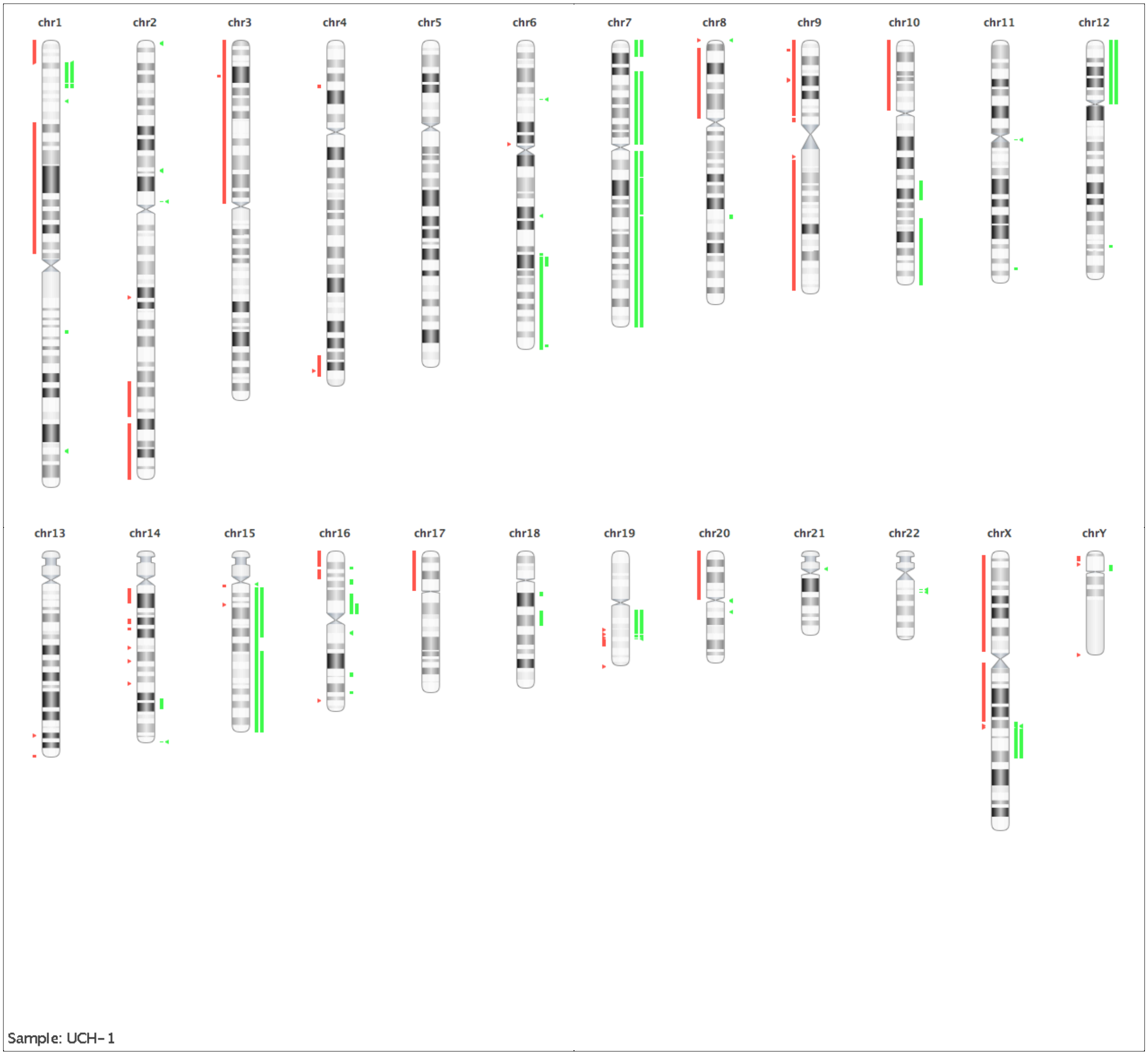
chr1
chr2
chr3
chr4
chr5
chr6
chr7
chr8
chr9
chr10
chr11
chr12
chr13
chr14
chr15
chr16
chr17
chr18
chr19
chr20
chr21
chr22
chrX
chrY
Sample: UCH-1

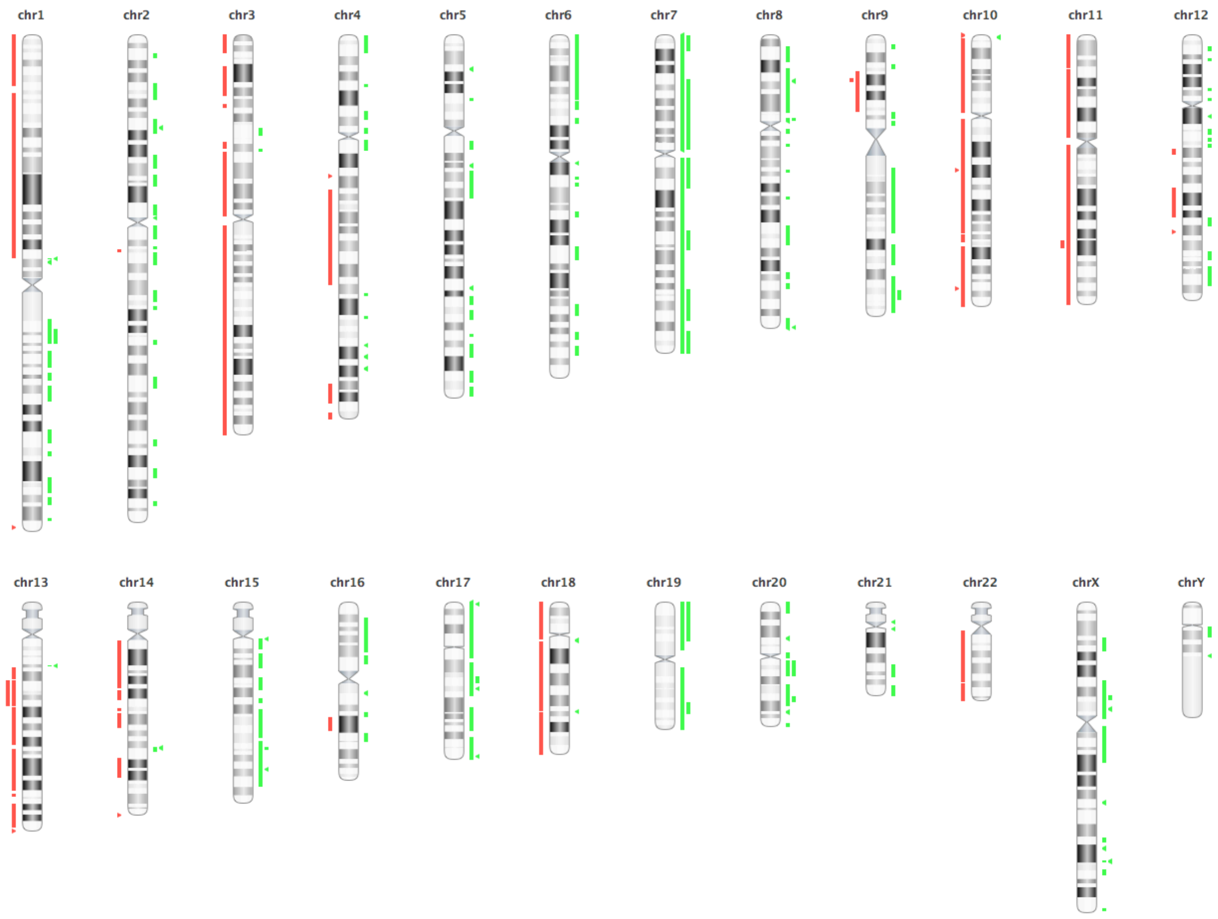
chr1
chr2
chr3
chr4
chr5
chr6
chr7
chr8
chr9
chr10
chr11
chr12
chr13
chr14
chr15
chr16
chr17
chr18
chr19
chr20
chr21
chr22
chrX
chrY

Sample: UCH-2

4 Genomic PCR for 5’ MTAP, 3’ MTAP, CDKN2A and DMRTA1 CNA

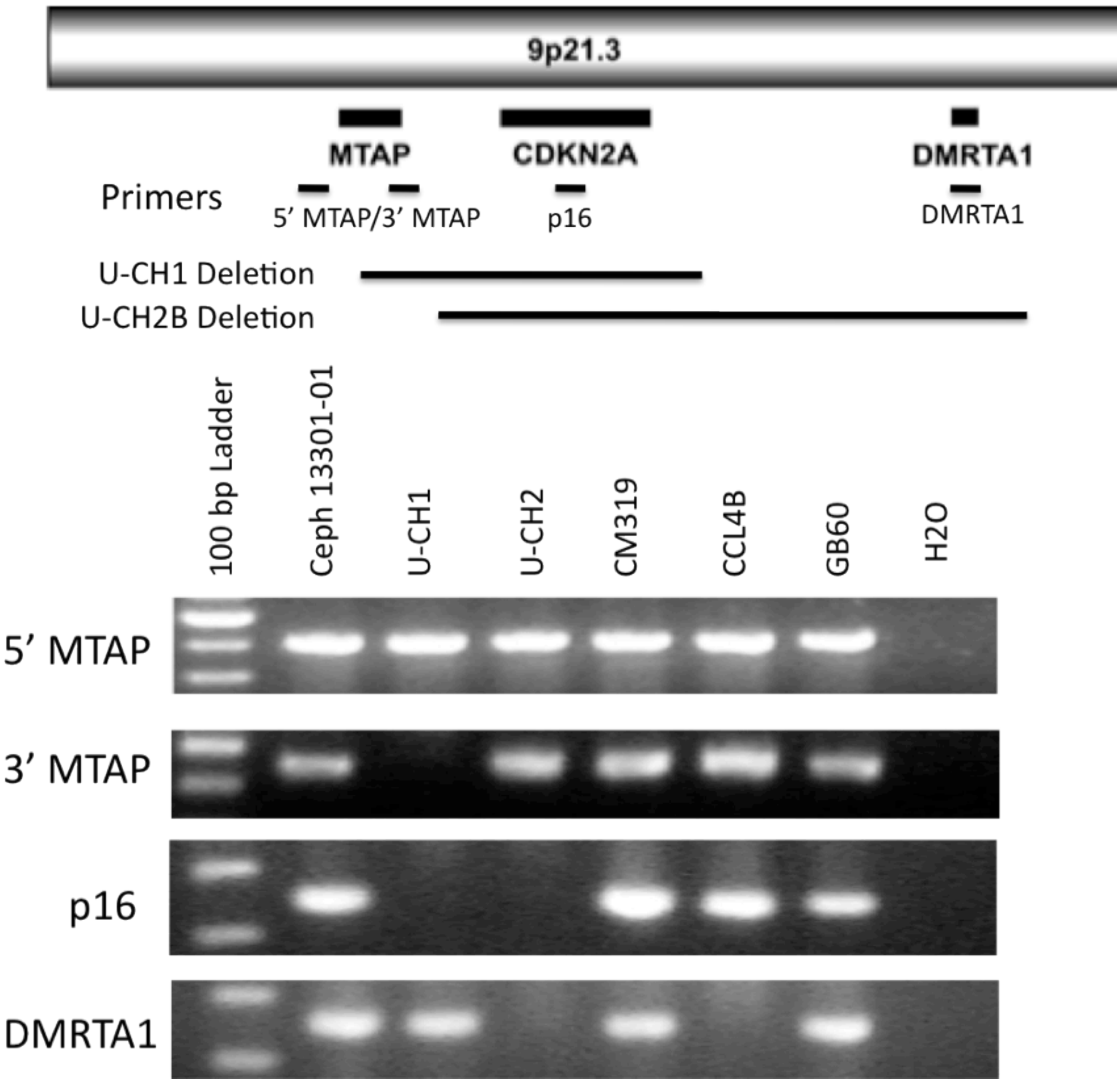

5. Array expression levels of PTEN, Vimentin and Keratin 8/19 in cell lines and select chordoma tumor samples.

Mas5 Analysis of Microarray expression of PTEN, Vimentin and Keratins in Chordoma cell lines and Tumors

				CELL LINES					Chordoma Tumors				
Gene symbol	Gene Title	Probe Set ID	Value reported	CCL4BI-Exp	GB60-Exp	CM-319-Exp	K001-Exp	U-CH1-Exp	U-CH2-Exp	Chordoma-H1-Exp	Chordoma-H2-Exp	Chordoma-H3-Exp	Chordoma-H4-Exp
**PTEN**													
PTEN	phosphatase and tensin homolog	204053_x_at	exp	2804.735	1840.909	3065.996	1200.142	48.10846	4151.174	412.4355	910.5419	597.5712	1247.374
PTEN	phosphatase and tensin homolog	204054_at	exp	408.9952	424.8596	988.3135	163.1766	10.88179	415.8873	27.77818	167.771	193.9418	168.8372
PTEN	phosphatase and tensin homolog	211711_s_at	exp	1496.392	1077.062	2023.792	333.697	40.54462	1616.937	215.8879	438.7045	335.8743	301.0594
PTEN	phosphatase and tensin homolog	222176_at	exp	3.477788	1.253621	8.55935	11.51182	19.23517	9.767094	12.55733	32.53456	25.02376	69.4368
PTEN	phosphatase and tensin homolog	204053_x_at	A-P	P	P	P	P	A	P	P	P	P	P
PTEN	phosphatase and tensin homolog	204054_at	A-P	P	P	P	A	A	P	A	P	A	A
PTEN	phosphatase and tensin homolog	211711_s_at	A-P	P	P	P	P	A	P	P	P	P	P
PTEN	phosphatase and tensin homolog	222176_at	A-P	A	A	A	A	A	A	A	A	A	A
**Vimentin (only one probe set shared on all platforms**		Probe Set ID		CCL4BI	GB60	CM-319	K001	U-CH1	U-CH2	Chordoma-H1	Chordoma-H2	Chordoma-H3	Chordoma-H4
VIM	vimentin	201426_s_at (Exp)	exp	65006.91	62029.39	8551.479	20238.59	11373.05	29177.5	28755.15	19509.95	34566.94	23101.81
		201426_s_at (A-P)	A-P	P	P	P	P	P	P	P	P	P	P
**Keratins**		Probe Set ID		CCL4BI	GB60	CM-319	K001	U-CH1	U-CH2	Chordoma-H1	Chordoma-H2	Chordoma-H3	Chordoma-H4
KRT8	Keratin 8	214399_s_at	exp	4.702704	13.23911	5.161353	39.42768	12.03273	25.06447	9.200591	9.397948	12.48427	12.32892
	keratin 8 /// keratin 8	209008_x_at	exp	143.0362	272.6878	3127.345	11324.18	10995.77	8831.125	3298.445	8120.014	8750.384	4757.128
	"keratin 8 /// similar to Keratin, type II cytoskeletal 8	216821_at	exp	12.99632	67.6403	66.19751	89.7683	94.29034	38.98143	131.0465	142.5518	150.0791	111.8744
	Keratin 8	214399_s_at	A-P	A	A	A	A	A	A	A	A	A	A
	keratin 8 /// keratin 8	209008_x_at	A-P	P	P	P	P	P	P	P	P	P	P
	KRT8	216821_at	A-P	A	A	A	A	A	A	A	P	A	A
		Probe Set ID		CCL4BI	GB60	CM-319	K001	U-CH1	U-CH2	Chordoma-H1	Chordoma-H2	Chordoma-H3	Chordoma-H4
KRT19	keratin 19	201650_at	exp	841.9418	36.92085	1.758223	20083.48	25434.49	27615.66	17602.82	19041.83	30790.4	24096.58
			A-P	P	A	A	P	P	P	P	P	P	P
